# Adeno-Associated Virus VP1u Exhibits Protease Activity

**DOI:** 10.3390/v11050399

**Published:** 2019-04-29

**Authors:** Justin J. Kurian, Renuk Lakshmanan, William M. Chmely, Joshua A. Hull, Jennifer C. Yu, Antonette Bennett, Robert McKenna, Mavis Agbandje-McKenna

**Affiliations:** Department of Biochemistry and Molecular Biology, Center for Structural Biology, The McKnight Brain Institute, University of Florida, Gainesville, FL 32610, USA; justinkurian@ufl.edu (J.J.K.); renuk@ufl.edu (R.L.); wchmely0812@ufl.edu (W.M.C.); the.hegemon@ufl.edu (J.A.H.); jennifer.yu@ufl.edu (J.C.Y.); dendena@ufl.edu (A.B.); rmckenna@ufl.edu (R.M.)

**Keywords:** Adeno-associated virus, AAV, protease, phospholipase-A_2_, PLA_2_

## Abstract

Adeno-associated viruses (AAVs) are being developed for gene delivery applications, with more than 100 ongoing clinical trials aimed at the treatment of monogenic diseases. In this study, the unique N-terminus of AAV capsid viral protein 1 (VP1u), containing a canonical group XIII PLA_2_ enzyme domain, was observed to also exhibit proteolytic activity. This protease activity can target casein and gelatin, two standard substrates used for testing protease function but does not self-cleave in the context of the capsid or target globular proteins, for example, bovine serum albumin (BSA). However, heated BSA is susceptible to VP1u-mediated cleavage, suggesting that disordered proteins are substrates for this protease function. The protease activity is partially inhibited by divalent cation chelators ethylenediaminetetraacetic acid (EDTA) and ethylene-bis(oxyethylenenitrilo)tetraacetic acid (EGTA), and human alpha-2-macroglobulin (A2M), a non-specific protease inhibitor. Interestingly, both the bovine pancreatic (group VIIA) and bee venom (group III) PLA_2_ enzymes also exhibit protease function against casein. This indicates that PLA_2_ groups, including VP1u, have a protease function. Amino acid substitution of the PLA_2_ catalytic motif (^76^HD/AN) in the AAV2 VP1u resulted in attenuation of protease activity, suggesting that the protease and PLA_2_ active sites are related. However, the amino acid substitution of histidine H38, which is not involved in PLA_2_ function, to alanine, also affects protease activity, suggesting that the active site/mechanism of the PLA_2_ and protease function are not identical.

## 1. Introduction

Adeno-associated viruses (AAVs) are members of the non-enveloped, single-stranded DNA packaging *Parvoviridae*. There are 13 non-human primate AAV serotypes, which range from 54 to 99% in capsid protein sequence identity [1,2]. In 2012, the European Medicines Agency approved “Glybera,” an AAV1 based capsid for the treatment of lipoprotein lipase deficiency [3] and in 2017 the United States Food and Drug Administration approved “Luxturna”, an AAV2 based vector for the treatment of Leber’s congenital amaurosis [4]. Currently, there are more than 100 clinical trials using AAV for the treatment of monogenic diseases. The cell/tissue binding and entry properties of the different AAVs guide the choice of serotype used in treatment applications. The AAV capsid is built from three viral proteins (VPs): VP1, VP2 and VP3, which assemble in a 1:1:10 ratio to form the T = 1 capsid consisting of 60 VPs (with approximately 5 VP1, 5 VP2 and 50 VP3) [5]. These VPs are overlapping in sequence, with VP1 containing a unique N-terminus referred to as the VP1-unique (VP1u) region. This VP1u region contains a group XIII phospholipase-A_2_ (PLA_2_) domain [6] shown to be essential for virus escape from endosomal/lysosomal compartments during trafficking to the nucleus [7,8]. Additional to the PLA_2_ activity, protease activity has also been associated with AAV capsids [9]. These studies showed that this protease activity was optimal at pH 7.4, attenuated at the lower pH of 5.5 and inhibited in the presence of a protease inhibitor cocktail. Protease function is common in many DNA and RNA viruses but these enzymatic domains are typically encoded separately from the viral structural proteins [10]. However, a few viral capsids have been shown to exhibit protease function, for example, Sindbis virus, which harbors a chymotrypsin-like serine protease motif [11].

In this study, AAV2 and AAV5 were used to identify the VP region responsible for the protease function, identify inhibitors/substrates and map residues associated with this function. The protease function was shown to reside within the VP1u region of the capsid and to be partially inhibited by metal chelators and the protein inhibitor alpha-2-macroglobulin (A2M). Additionally, the AAV2 protease was observed to have a preference for disordered protein substrates. Significantly, known PLA_2_ enzymes, for example, the bovine pancreatic and bee venom PLA_2_ enzymes also possess protease function, indicating that PLA_2_ domains have additional functional roles not previously reported. Amino acid substitution for residues in the VP1u domain associated with PLA_2_ catalysis resulted in impaired protease function and a corresponding decrease in virus transduction, suggesting a mechanistic link between PLA_2_ domains and protease activity.

## 2. Materials and Methods

### 2.1. AAV2 Plasmid Mutagenesis

The QuikChange Lightning PCR site-directed mutagenesis kit (Agilent, Santa Clara, CA, USA) was utilized to make mutations onto an AAV2 template plasmid conferring ampicillin resistance (pXR2) [12]. Following digestion of template DNA by Dpn-1 enzyme, One Shot™ TOP10 Chemically Competent *E. coli* (Thermo Fisher Scientific, Waltham, MA, USA) were transformed using the PCR mix and incubated for 1 h in an orbital shaker set at 37 °C after the addition of Super Optimal Broth with Catabolite repression (S.O.C.) medium (Thermo Fisher Scientific). The *E. coli* cultures were plated onto LB-Agar containing ampicillin at a concentration of 50 µg/mL and incubated at 37 °C for a minimum of 12 h, followed by selection of individual bacterial colonies for a minimum of 12 h growth at 37 °C in lysogeny broth (LB) medium containing ampicillin at a concentration of 50 µg/mL. Plasmid DNA from these cultures was purified using a QIAprep Spin Miniprep Kit (Qiagen, Hilden, North Rhine-Westphalia, Germany) and mutations were confirmed by Sanger sequencing (Genewiz, South Plainfield, NJ, USA). Larger quantities of plasmid were produced by inoculating 500 mL of LB medium containing ampicillin at a concentration of 50 µg/mL with bacterial glycerol stocks containing plasmids with the desired mutations and growing cultures for a minimum of 12 h at 37 °C. Plasmid DNA for transfection was purified using a PureLink™ HiPure Plasmid Filter Maxiprep Kit (ThermoFisher Scientific). The rAAV2 variants made were ^76^HD/AN, H95A, H38A, D69A and D97A. AAV2 VP variant VLPs were generated by site directed mutagenesis (Agilent) of the AAV2 plasmid pFBDVPm11 as previously reported [13]. Briefly, the VP1 and VP2 start codons were mutated to GCG to generate AAV2-VP13, AAV2-VP23 and AAV2-VP3 only constructs. The wild-type AAV2, AAV5 and mutant plasmids were used to generate recombinant VLPs in *Sf9* cells via the Bac-to-Bac expression system (Thermo Fisher Scientific) according to the manufacturer’s protocol as previously reported [14].

### 2.2. Production and Purification of rAAV Capsids

HEK293 cells were grown on 15 cm plates in Dulbecco’s Modified Eagle’s Medium (Sigma-Aldrich, St. Louis, MO, USA) containing 10% *v/v* fetal bovine serum (Sigma-Aldrich) and 1% Antibiotic-Antimycotic (Thermo Fisher Scientific) to a confluency of 80%, followed by transient transfection using the pXR2 or pXR5, pHelper and pTR-UF3-Luc plasmids in an equimolar ratio [15,16,17], with polyethylamine (PEI) utilized as the transfection reagent. Transfected cells were incubated at 37 °C and 5% CO_2_ for 72 h and subsequently harvested. Cells were pelleted by centrifugation at 1590× *g* for 30 min and resuspended in 1× TD buffer (1× PBS with 1 mM MgCl_2_ and 2.5 mM KCl). Ten percent *w/v* PEG 8000 was added to the supernatant from the cell harvest and incubated at 4 °C for 12 h with mechanical stirring to precipitate virus. The PEG 8000-supernatant mix was centrifuged at 14,300× *g* for 90 min to pellet precipitated virus. The resulting supernatant was discarded, and the pellet was resuspended in 1× TD. The concentration of NaCl in the resuspended cell pellet was adjusted to 1 M and the cell pellet was lysed by a series of three freeze-thaw cycles in a liquid nitrogen bath. Following the final freeze cycle, 250 U of Benzonase nuclease (MilliporeSigma, Burlington, MA, USA) was added to both the lysed cell pellet and the resuspended pellet from PEG 8000 precipitation and incubated at 37 °C for 30 min in order to remove any unpackaged DNA on the capsid surface. The pellets were combined and clarified by centrifugation at 12,100× *g*. The supernatant was loaded onto a discontinuous iodixanol (Optiprep–Sigma-Aldrich) density gradient with layers at 15, 25, 40 and 60% as previously described [18] and centrifuged at 350,000× *g* for 1 h at 12 °C. Each gradient tube was collected in 1 mL fractions and analyzed for the presence of packaged DNA by qPCR. Combined fractions of either full (DNA containing) or empty (no DNA) particles were diluted by 10-fold in 1× TD buffer and loaded onto AVB Sepharose columns (GE Healthcare Life Sciences, Marlborough, MA, USA) at a rate of 1 mL/min. Columns were washed with 10 mL of 1× TD buffer at a rate of 1 mL/min, followed by elution with 0.1 M Glycine-HCl at pH 2.7 fractionated into 500 µL fractions. Each fraction was immediately combined with 100 µL of 1 M Tris buffer at pH 10.0 to neutralize the acidic elution buffer conditions. Peak fractions, as indicated by UV absorbance at 280 nm, were combined and buffer exchanged three times into buffer containing 20 mM HEPES, 20 mM MES, 20 mM NaAc, 150 mM NaCl using an Apollo 7 mL 150 kDa molecular weight cutoff centrifugal concentrator (Orbital Biosciences, Topsfield, MA, USA). The final buffer exchange cycle used 20 mM HEPES, 20 mM MES, 20 mM NaAc, 150 mM NaCl and 5 mM CaCl_2_ (Universal Buffer) [19] and stored at 4 °C after assessing concentration and purity via sodium dodecyl sulfate–polyacrylamide gel electrophoresis (SDS-PAGE).

### 2.3. Production and Purification of AAV Virus-Like Particles (VLPs)

Infected cells were harvested at 72 h, resuspended in 1× TD, lysed by three freeze-thaw cycles with Benzonase nuclease (MilliporeSigma) treatment after the third freeze-thaw cycle as described above for rAAV2 samples. The supernatant was treated with 10% PEG 8000 and precipitated VLPs were harvested by centrifugation of the PEG-supernatant mixture at 14,300× *g* and the precipitate resuspended in 1× TD, followed by Benzonase treatment as above. The lysed cells and resuspended PEG precipitated capsids from the supernatant were combined and clarified via centrifugation at 12,100× *g*. The clarified lysate was loaded onto a discontinuous iodixanol gradient (15, 25, 40 and 60%) and centrifuged at 350,000× *g* for 1 h at 12 °C. Following ultracentrifugation, the 40 and 40/25 interface (containing the VLPs) were collected and diluted in a 20 mM Tris, 15 mM NaCl buffer (pH 8.5). The combined fractions were loaded onto a HiTrap Q XL column (GE Healthcare Life Sciences) and eluted with buffer containing 20 mM Tris-HCl, 500 mM NaCl (pH 8.5). Peak fractions were combined and concentrated using an Apollo 150 kDa MW cutoff centrifugal concentrator (Orbital Biosciences) and buffer-exchanged into Universal Buffer. Samples of wild type (wt) AAV2 and AAV5 empty capsids produced in HEK293 cells were also purified by iodixanol centrifugation, the 40 and 40/25 interface collected, further purified by ion-exchange chromatography and dialyzed into Universal Buffer at pH 7.4, as per the protocol described for the VLPs. All samples were stored at 4 °C prior to use.

### 2.4. Negative-Stain Transmission Electron Microscopy

The integrity of all purified virus samples was confirmed using transmission electron microscopy. For each sample, 2 µL of purified virus was applied to carbon-coated copper grids (Electron Microscopy Sciences, Hatfield, PA, USA) for 2 min followed by wicking of excess buffer with filter paper (GE Healthcare Life Sciences). Grids were washed with 10 µL of filtered water, excess liquid wicked off and then stained with 10 µL 1% uranyl acetate for 10 s. Stain was removed with filter paper and grids were imaged on a 120 keV Tecnai Spirit (Thermo Fisher Scientific).

### 2.5. Substrate Preparation and Protease Assays

Casein substrate (Sigma-Aldrich) was prepared by dissolving 50 mg of casein powder in 5 mL 1 M NaOH and dilution of the mixture with filtered dH_2_O to a final volume of 50 mL. Following complete solubilization, the casein was dialyzed against 4 L of Universal Buffer prepared at pH 7.4. Following the final dialysis step, the casein was filtered using a 2 µm filter and stored at 4 °C until used. Gelatin substrate (Sigma-Aldrich) was prepared by reconstitution in filtered deionized water and mixing at 37 °C until solubilized, followed by storage at 4 °C. Several protein samples were purchased and reconstituted in filtered dH_2_O prior to use in protease assays: bee venom PLA_2_, bovine pancreatic PLA_2_, A2M (Sigma-Aldrich) and trypsin (Thermo Fisher Scientific). Bovine serum albumin (BSA) was also purchased (Thermo Fisher Scientific), reconstituted in filtered dH_2_O and incubated at 4 °C (native BSA) or at 100 °C for 30 min (heated BSA), followed by incubation at 4 °C for at least 1 h prior to further use. Protease assays were conducted by measuring breakdown of a substrate by SDS-PAGE analysis of samples incubated at 37 °C for specified time points. Protease inhibition studies used either an inhibitor cocktail (Pierce Protease Mini Tablets with and without EDTA), EDTA/EGTA or A2M that was added prior to assay initiation. Metal chelation inhibition assays used EDTA or EGTA at concentrations of either 4 mM or 100 mM EDTA in presence of AAV2 capsids at a concentration of 46 nM and 5 μg total amount of casein substrate. A2M inhibition studies used A2M at a concentration of 460 nM, AAV2 capsids at 46 nM and 5 μg total amount of casein substrate. Protease assays with BSA used 5 μg of native BSA (N-BSA) or heated BSA (H-BSA) in conjunction with AAV2 capsids at 15 nM or a trypsin control at 22 μM. In protease assays comparing different PLA_2_ enzymes, bee venom PLA_2_, bovine pancreatic PLA_2_ and bovine serum albumin (BSA) were at a concentration of 5 μM and AAV2 capsids were at a concentration of 15 nM. 2.5 μg of casein substrate was present in each reaction. Protease assays comparing wt AAV2 to VP1u variant capsids utilized virus samples at 15 nM and total of 2.5 μg casein per reaction. Quantitation of substrate degradation was performed using gel densitometry with the Image Studio Lite software (Li-COR Biosciences, Lincoln, Nebraska, USA) and statistical significance was determined via single factor ANOVA analysis, with annotation conventions assigned per the following: ns (*p* > 0.05), * (*p* ≤ 0.05), ** (*p* ≤ 0.01), *** (*p* ≤ 0.001), **** (*p* ≤ 0.0001). Error bars on all figures represent standard error of the mean. Protease assays with AAV5 samples (at a concentration of 80 nM) were conducted using the Pierce Colorimetric Protease Assay Kit (Thermo Fisher Scientific) in accordance with manufacturer protocols; the lyophilized succinylated casein substrate was reconstituted in Universal Buffer at pH 7.4 or 5.5, instead of the supplied borate assay buffer. All experiments with quantification and statistical analysis applied were performed in triplicate.

### 2.6. PLA_2_ Assays

PLA_2_ activity of wt AAV2 and variant viruses (30 nM) were assessed using a sPLA_2_ assay kit (Cayman Chemical, Ann Arbor, MI, USA) in accordance with the manufacturer’s protocol. The assay plate was incubated at 37 °C for 72 h and absorbance readings at 414 nm were recorded and statistical significance was determined via single factor ANOVA analysis, with annotation conventions assigned per the following: ns (*p* > 0.05), * (*p* ≤ 0.05), ** (*p* ≤ 0.01), *** (*p* ≤ 0.001), **** (*p* ≤ 0.0001). Error bars on all graphs represent standard error of the mean. This assay was conducted at pH 8.0, the condition provided in the kit.

### 2.7. Cellular Transduction Assays

HEK293 cells were seeded in a 96-well plate and grown to 50% confluency (2.5 × 10^4^ cells/well) at 37 °C and 5% CO_2_. Media was removed from wells and virus added at a multiplicity of infection (MOI) of 10^4^ in a volume of 30 μL (containing virus sample plus serum-free DMEM). Virus sample titer was determined by quantitative PCR as previously described [20]. For assays with A2M, 10 μg of A2M was incubated with 2.5 × 10^8^ viral genomes (vg) of AAV2 for 24 h at either 4 or 37 °C. Plate was incubated for 30 min at 37 °C and 5% CO_2_, followed by addition of 70 μL of DMEM containing 10% fetal bovine serum and 1% Antibiotic-Antimycotic. Plate was further incubated at 37 °C and 5% CO_2_ for 48 h and luciferase expression was determined using a Luciferase Assay System (Promega, Madison, WI, USA). Prior to acquisition of luciferase activity, each well on the plate was gently washed three times with 50 μL of 1× PBS followed by cell lysis with 50 μL of a 1× preparation of the supplied 5× lysis buffer. Cell lysis proceeded for 20 min at room temperature, followed by transfer of 30 μL of lysate from each well into a new 96-well plate with opaque housing between individual wells (suitable for the determination of sample luminescence). Thirty microliters of luciferase assay reagent was added to each well and luminescence from each sample was acquired with a Synergy HTX Multi-Mode plate reader (BioTek, Winooski, VT, USA), and statistical significance was determined via single factor ANOVA analysis, with annotation conventions assigned per the following: ns (*p* > 0.05), * (*p* ≤ 0.05), ** (*p* ≤ 0.01), *** (*p* ≤ 0.001), **** (*p* ≤ 0.0001). Error bars on all graphs represent standard error of the mean.

### 2.8. Structural Modeling and Sequence Alignments

Structural models of the AAV2 VP1u (137 amino acids) and AAV5 VP1u (136 amino acids) domain were generated using the RaptorX protein prediction server [21]. Crystal structures of bee venom PLA_2_, bovine PLA_2_ and human sPLA_2_ were accessed and downloaded from RCSB PDB (PDB IDs: 1POC, 1UNE and 1KQU, respectively). Images were rendered using UCSF Chimera [22] and RMSD values calculated using PyMOL [23]. Sequence alignments were performed using the Clustal Omega webserver [24].

## 3. Results and Discussion

### 3.1. AAV Protease Function Is VP1u Dependent, Active at Physiological pH, Calcium Enhanced and Inhibited by a Protein Protease Inhibitor

**Figure 1:**
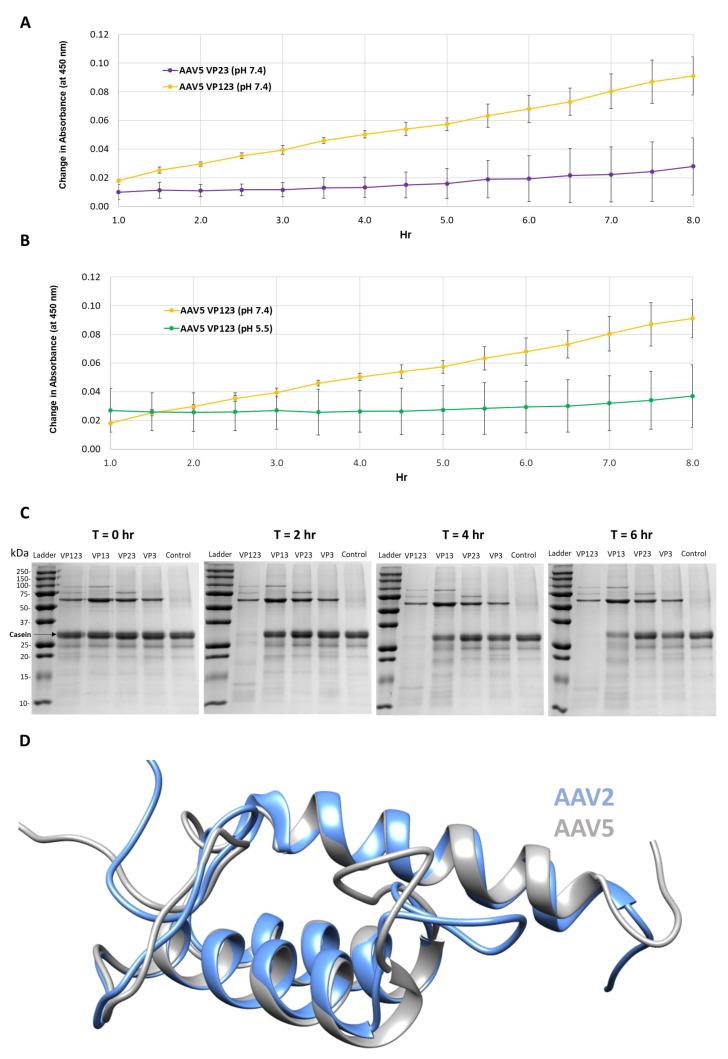
Protease activity is VP1 and neutral pH dependent. (**A**) Colorimetric readout (*y*-axis) over time (hr, *x*-axis) for a casein substrate degradation by AAV5 capsids assembled from VP1, VP2 and VP3 or only VP2 and VP3. (**B**) Same as in (**A**) but for AAV5 VP1, VP2 and VP3 capsids at pH 7.4 and 5.5. (**C**) SDS-PAGE of AAV2 VLPs, containing the VPs shown, incubated with a casein substrate at 37 °C for 2, 4 and 6 h. Observations confirm the need to have VP1 and physiological pH for activity. (**D**) Superposition of AAV2 (blue) and AAV5 (gray) VP1u models. *N*-terminal domains of models with predicted disorder not shown.

The protease activity associated with AAV serotypes has been previously reported [9]. Thus, a primary goal of this study was to locate and confirm the region within the VPs or capsid responsible for this function. Protease assays with wt AAV5 capsids containing all three VPs, VP1, VP2 and VP3 or only VP2 and VP3 showed that capsids missing VP1 had no protease activity at pH 7.4 (Figure 1A). In addition, protease activity was negligible for VP1, VP3 and VP3 containing capsids at pH 5.5 as previously reported [9] (Figure 1B).

Protease assays with AAV2 VLP variants containing different combinations of VP: VP1, VP2 and VP3; VP1 and VP3; VP2 and VP3; and VP3 only, confirmed the need for VP1 for proteolytic activity (Figure 1C). Only the VLPs containing VP1 (lanes 1 and 2 after the molecular weight marker lane) showed the degradation of the casein substrate. The requirement of VP1 and hence VP1u in both AAV2 and AAV5 for the observed protease activity suggests that this domain shares sequence and structure similarity between the viruses (Figure 1D). Consistently, they share 68% amino acid identity and the 3D models predicted for ordered regions (residues 48–137 in AAV2 and 47–136 in AAV5), imply a three-helix bundle, with a calculated Cα RMSD of 0.6 Å (Figure 1D). These observations indicate that the VP1u region has a second enzymatic function in addition to the previously described PLA_2_ activity [8]. However, the requirement of physiological and not acidic conditions for protease activity (Figure 1B) is in contrast to the PLA_2_ activity that requires acidic conditions [25]. This protease function does not appear to act on other capsids or other VP1us in the context of the capsid, because there is no observable reduction in the abundance of the three VPs at the end of the reaction (Figure 1C). This is contrary to the characteristics of other proteases, for example, trypsin, in which the enzyme also eventually becomes its own substrate [26,27]. The current thinking is that VP1u is located in the capsid interior and becomes externalized during the endo/lysosomal pathway to enable the PLA_2_ function required for capsid escape en route to the nucleus for genome replication. Significantly, the protease activity within the VP1u occurs without applying an external treatment, for example, heat, to the capsid to “externalize” this VP region as reported to be required for PLA_2_ activity in assays outside a cell [28]. This suggests that the VP1u domain is dynamic in its capsid positioning and able to partially externalize for function in the absence of external treatments, including acidic pH and/or increased temperature conditions proposed to induce structural rearrangement of the capsid resulting in VP1u externalization [29,30,31,32].

**Figure 2:**
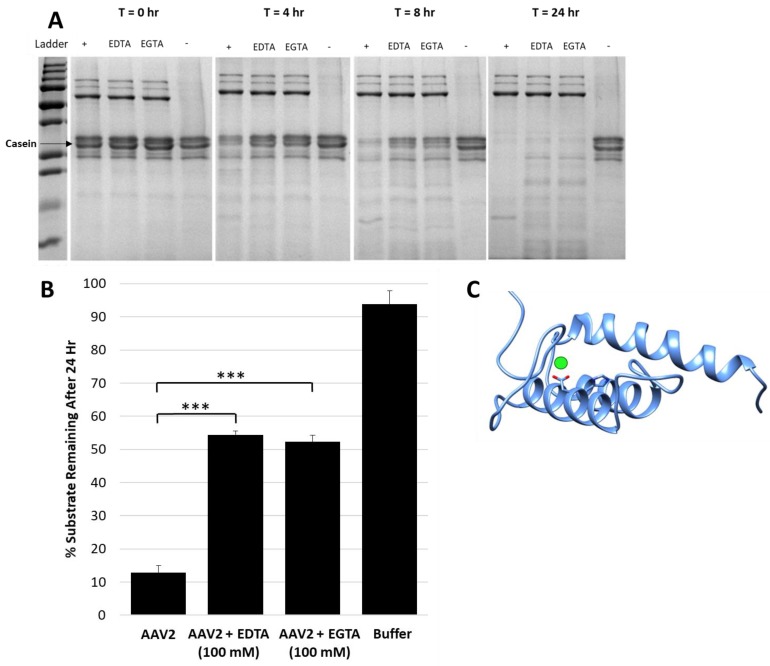
Metal chelators EDTA and EGTA reduce protease activity. (**A**) SDS-PAGE showing casein proteolysis by AAV2 in presence of 4 mM EDTA or EGTA. “+” indicates control with no inhibitor and “-” indicates control with no AAV2, that is, casein alone. The lane with AAV2 only (“+”) shows fastest decrease in casein with time. (**B**) Quantification of casein proteolysis by AAV2 in presence of either 100 mM EDTA or 100 mM EGTA. The *y*-axis indicates the amount of remaining protein after 24 h. (**C**) Predicted RaptorX structure model of AAV2 VP1u. PLA_2_ catalytic residues H75 and D76 are in orange colored sticks, the predicted site of bound calcium is denoted by a green sphere next to D76. A predicted disordered portion of AAV2 VP1u N-terminal residues is not shown. Statistical significance as indicated by asterisk annotations (*) are described in Material and Methods section.

A protease inhibitor cocktail with the divalent cation chelator EDTA was required to inhibit the AAV2 protease function, in addition to the observation that EDTA or EGTA alone reduced activity (Figure 2A,B). Assays with an excess of either of these chelators (100 mM, 10^7^-fold molar excess to capsids) only inhibited ~50% of activity after 24 h (Figure 2B). This observation suggests that the catalytic activity can proceed independent of divalent cations. The VP1u of parvoviruses is predicted to have a calcium binding region that is located within residues 45–65 in AAV2 and is essential for PLA_2_ activity [8]. A VP1u model predicts calcium bound in a loop adjacent to the PLA_2_ active site HDXXY motif (Figure 2C). The prediction is that calcium binding plays a stabilization role for VP1u and therefore its PLA_2_ and protease domain, because it lacks the disulfide bond interactions present in other PLA_2_ domains [6]. The results suggest that the proteolytic function of the AAV2 VP1u differs in mechanism from its calcium-dependent PLA_2_ activity.

**Figure 3:**
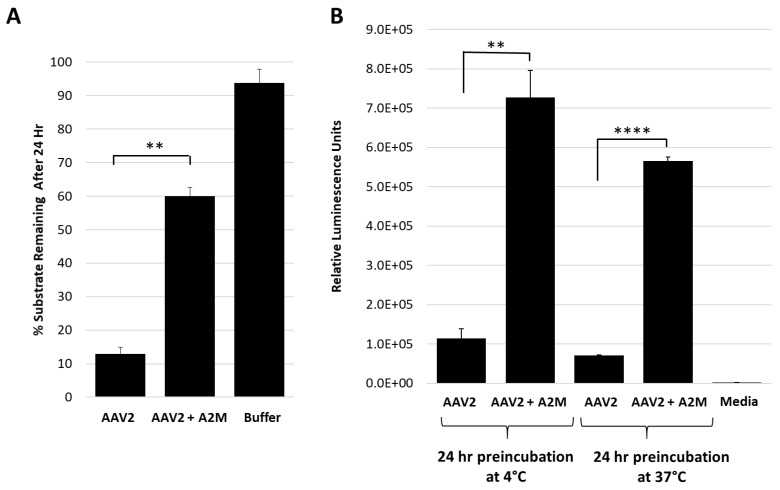
Alpha-2-macroglobulin inhibits protease function and correlates with increased cellular transduction. (**A**) AAV2 protease function inhibition in presence of A2M. The *y*-axis shows the amount of casein substrate remaining after 24 h at the conditions indicated (*x*-axis). (**B**) Luciferase gene expression (*y*-axis in relative luminescence units) for AAV2 with or without A2M (*x*-axis) prior to cell infection. Statistical significance as indicated by asterisk annotations (*) are described in Material and Methods section.

The test of the general protease inhibitor, A2M, against AAV2 also resulted in ~50% loss in activity (Figure 3A). For these studies, the A2M was present at a 10-fold molar excess to the AAV2 capsid and achieved levels of inhibition comparable to EDTA/EGTA at 10^7^-fold molar excess (Figure 2B). A2M contains a bait region into which enzymes are captured and their function inhibited [33]. A cellular transduction assay, conducted to determine if the inhibition of the proteolytic activity of AAV2 coincides with an effect on infectivity, showed a 5.5- to 7-fold increase in luciferase gene expression level for sample preincubated for 24 h at 37 and 4 °C, respectively (Figure 3B). The 37 and 4 °C treatments recapitulate protease assay and storage conditions, respectively. It is possible that improved cellular internalization of A2M via endocytosis by selective cell surface receptors [34,35] may be responsible for this increase in AAV2 transduction. Alternatively, this increase in transduction may be attributable to enhanced cellular growth by usage of A2M as an energy source similar to the effects of fetal bovine serum [36], thus also resulting in an improved luciferase gene expression. Significantly, the interaction between the serum protein human serum albumin (HSA) and AAVs was reported to increase cell binding and transduction [37]. A similar mechanism may be in effect for the observed increase in transduction for the AAV samples incubated with A2M.

### 3.2. The Targets for AAV2 Protease Activity Are Disordered or Unfolded Proteins

**Figure 4:**
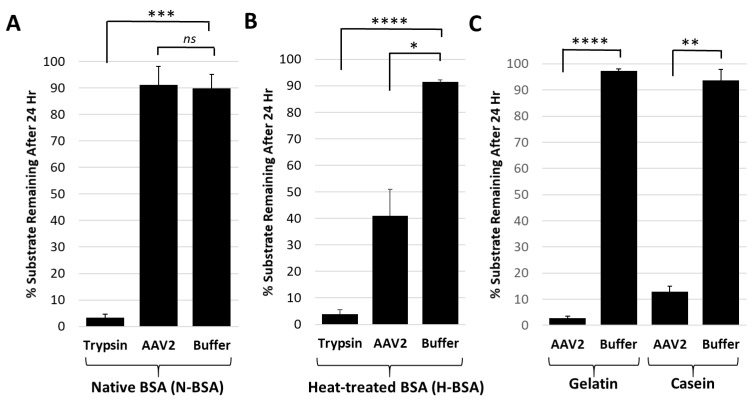
The AAV2 VP1u protease preferentially degrades disordered proteins. (**A**) Quantification of trypsin or AAV2 protease activity against native BSA (N-BSA). The *y*-axis shows the percentage of substrate remaining after 24 h under the conditions indicated in the *x*-axis. (**B**) Quantification of trypsin or AAV2 activity against heated BSA (H-BSA). Axes are as defined in (A). (**C**) Quantification of AAV2 activity against casein and gelatin. Axes are as defined in (A). Data for casein was shown in Figure 2. Statistical significance as indicated by asterisk annotations (*) are described in Material and Methods section.

Casein and gelatin substrates used in the protease assays have no defined tertiary structure and are disordered in nature. Casein is micellar in solution [38] and gelatin is a mix of heterogenous peptides generated from collagen hydrolysis [39]. Protease assays with both native and heat-treated BSA were conducted to test the possibility that disordered proteins are the target for the AAV2 VP1u protease function. The heat-treated BSA was degraded by AAV2 VP1u while native BSA was not (Figure 4A,B). In contrast, trypsin, used as a positive control protease, showed equivalent degradation of both the native and heat-treated BSA samples (Figure 4A,B). Prior studies have shown that heating of BSA results in an increase in the percentage of disordered secondary structure elements [40]. Thus, combined with the casein and gelatin substrate data showing degradation (Figure 1 and Figure 4C), these observations suggest that the cellular targets for AAV2 protease activity are either intrinsically disordered proteins and/or proteins that possess unstructured regions.

Many cellular proteins fit the criteria of being intrinsically disordered or containing disordered regions, including transcription factors, cellular signaling proteins and nucleoporins [41]. Nucleoporins possess disordered phenylalanine-glycine repeats [42] and are major components of the nuclear pore complex (NPC). The parvoviruses traffic to the nucleus for genome replication and are predicted to interact with the NPC to facilitate entry [43]. Thus, it is possible that disordered proteins within the NPC are a target of the AAV2 protease function. While regions of the AAV2 VP1u are predicted to be disordered (Figure 2C) [30], as already stated above, self-degradation of VP1 in the context of the capsid is not evident during the 24 h time scale of the assays, possibly because the assembled capsid stabilizes the VP1u domain into a more ordered state compared to the 3D model generated (Figure 2C). In addition, it is possible that the ~5 individual VP1 monomers in the capsid and hence their VP1u, may be too distantly spaced and therefore incapable of inter-monomer cleavage.

### 3.3. Protease Function May Be a General Activity for PLA_2_ Enzymes

**Figure 5:**
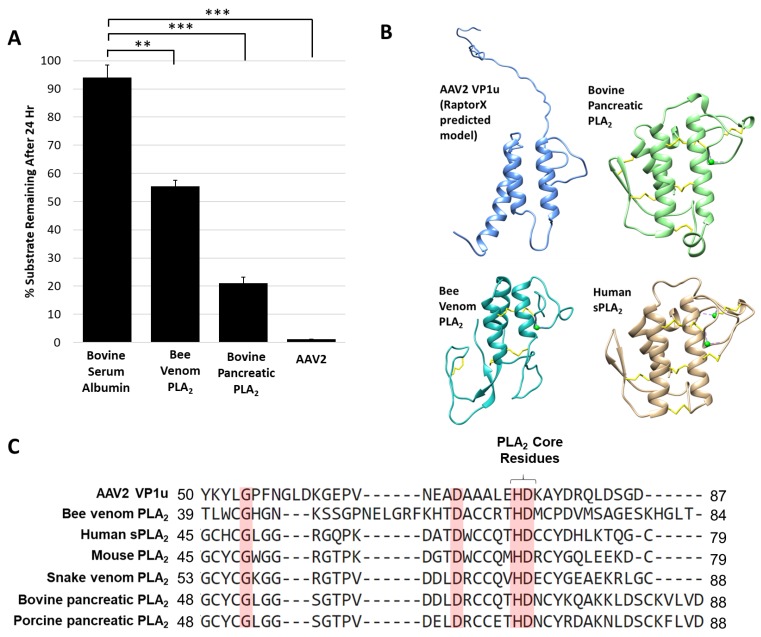
Protease activity is a general property of PLA_2_ enzymes: (**A**) Quantification of casein substrate degraded by bee venom PLA_2_, bovine pancreatic PLA_2_ and AAV2. The *y*- and *x*-axes are as described in Figure 4. (**B**) Predicted structure of AAV2 VP1u compared to bee venom PLA_2_ (PDB ID: 1POC), bovine pancreatic PLA_2_ (PDB ID: 1UNE) and human sPLA_2_ (PDB ID: 1KQU). (**C**) Partial amino acid sequence alignment of PLA_2_ enzymes (conserved residues are highlighted in red). Statistical significance as indicated by asterisk annotations (*) are described in Material and Methods section.

The AAV protease activity resides within VP1u (Figure 1) that also contains a PLA_2_ domain, thus known non-viral PLA_2_ enzymes were tested for their ability to degrade proteins. The protease activity assay for two PLA_2_ enzymes commonly used as positive controls, bee venom and bovine pancreatic, showed degradation of a casein substrate, albeit at a reduced level, 5- and 2-fold, respectively, compared to AAV2 (Figure 5A). This reduced activity was despite the use of a 50 molar higher concentration of the non-viral enzymes compared to AAV2, suggesting an enhanced activity with a functional yet to be described role in the virus. Interestingly, previous studies performed with human sPLA_2_ and porcine PLA_2_ indicated that these and other PLA_2_ enzymes have the ability to cleave apolipoprotein A-1 when the substrate was not bound to lipids [44]. The authors of this study proposed that the proteolytic function observed may also be PLA_2_ independent, because metal chelation inhibited PLA_2_ function but was not enough to eliminate protease activity, as was also observed for the AAV2 VP1u (Figure 2). The VP1u of AAV2 shares sequence and structural homology with non-viral PLA_2_s despite a lack of disulfide bonds (Figure 5B,C). This includes the residues required for PLA_2_ activity and calcium binding. The structures include a 3-helix bundle with connecting loops, including the calcium binding loop (Figure 5B). These observations suggest convergent evolution in structure to aid function for these enzymes.

**Figure 6:**
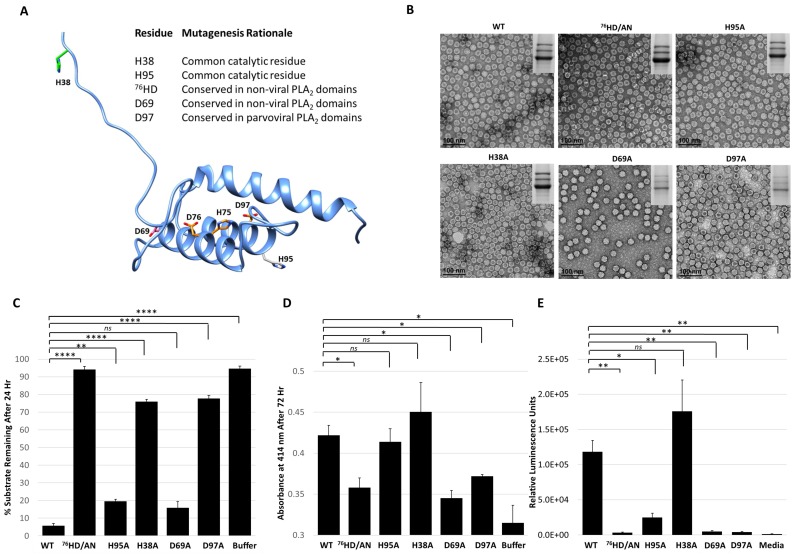
Functional phenotypes of AAV2 VP1u variants. (**A**) Location of amino acid substitutions in the AAV2 VP1u listed and shown in stick representation on a RaptorX model. Residues colored in orange represent those with PLA_2_ and protease defects when substituted, pink for defect to PLA_2_ only, green for defect to protease only and gray for no defect to either PLA_2_ or protease function. (**B**) Negative-stain EMs of wt AAV2 and variant capsids, confirming the capsid assembly of the viruses. SDS-PAGE gel insert shows expression of VP1, VP2 and VP3; (**C**) Quantification of the proteolysis of a casein substrate by wt AAV2 and the variants listed (*x*-axis) after 24 h. The *y*-axis is as described in Figure 2. (**D**) PLA_2_ activity of wt AAV2 and variants. The *y*-axis depicts the level of lipid modification after 72 h at pH 8.0 and *x*-axis lists the samples tested. (**E**) Luciferase reporter activity expression (RLU) for wt AAV2 and variants. The *y*-axis depicts the relative luminescence units and *x*-axis lists the samples tested. Statistical significance of asterisk annotations (*) are described in Material and Methods section.

Site directed mutagenesis was used to better characterize the determinants of AAV2 VP1u protease function based on conserved sequence and structural homology with other PLA_2_ enzymes (Figure 5B,C and Figure 6A). In addition to the “HD” PLA_2_ motif, a sequence alignment highlighted other identical residues, including a glycine residue located in the calcium-binding loop (residue 54) and an aspartic acid (residue D69) (Figure 5C), which was selected for mutagenesis. Previously, it had been shown for AAV2 that deletion of the VP1u calcium-binding loop containing the conserved glycine was detrimental to PLA_2_ function [8] but a potential role for the conserved aspartic acid (D69, VP1 AAV2 numbering) has not been delineated. Another residue mutated was D97, conserved in parvovirus PLA_2_ domains and previously shown to result in defective transduction when substituted [45]. Finally, the two remaining histidine residues in VP1u, H38 and H95, were modified because histidines are commonly associated with enzyme active sites [46] and often studied for their role in catalysis (Figure 6A).

The five VP1u variants made, H38A, D69A, ^76^HD/AN, H95A and D97A, all express VP1, VP2 and VP3 in the expected ratio of VP1:VP2:VP3 and assemble capsids (Figure 6B). The variants displayed various phenotypes with respect to protease and PLA_2_ activity and transduction efficiency. The H38A, ^76^HD/AN and D97A variants were observed to have an 8–10-fold decrease in protease activity compared to wt AAV2, with the most attenuation associated with the ^76^HD/AN variant (Figure 6C). While D69A is active for protease function, it along with the ^76^HD/AN and D97A variants displayed reduced PLA_2_ activity and cellular transduction (~50-fold reduction in RLU compared to wt AAV2) (Figure 6D,E). In contrast, the H38A variant, with a protease defect, has no PLA_2_ and transduction defect (Figure 6E). Since H38 is not conserved amongst non-viral PLA_2_ enzymes (Figure 5C) or with other AAV serotypes, the mode by which it impacts protease function is not readily apparent. Although the sequence region around H38 is predicted to be structurally disordered (Figure 6A), the effect of this residue on protease function suggests that it could be positioned closer to the folded, globular domain with the alpha helices. The D69 residue is conserved between AAV2 and non-viral PLA_2_s, and its amino substitution did not affect protease activity but did result in reduced PLA_2_ function and reduced cellular transduction compared to wt AAV2. This suggests that D69 is involved in PLA_2_ function and may be more critical to regulating this enzymatic function than previously understood (Figure 6E). The aspartic acid in the “HD” motif is predicted to coordinate the calcium ion interacting with the putative calcium binding loop of VP1u (Figure 2C). Thus, the substitution of this key amino-acid could perturb the calcium binding ability of the VP1u, resulting in a more defective protease activity phenotype as is observed for the ^76^HD/AN variant, possibly due to reduced stability of the protein fold in absence of metal binding. The H95A variant had no effect on protease or PLA_2_ function compared to wt AAV2 but did result in a decrease in cellular transduction, indicating that this phenotype is mediated by other factors. Interestingly, H95 is conserved in AAV VP1s but has no structurally analogous equivalent in other non-viral PLA_2_s.

The observations with the five variants tested indicate that while a protease function exists in VP1u, there is significant overlap between the residues involved and those enabling the PLA_2_ function within this capsid region. However, these residues are not identical and the data show that the PLA_2_ function is the most essential for cellular transduction. A His-Asp-Ser catalytic triad is common in a wide variety of proteases but numerous variations to this common motif have also been seen, such as His-His-Ser (cytomegalovirus protease) and Ser-Lys (bacterial type I signal peptidase) [47]. Yet, there are no conserved serine residues among the different PLA_2_ enzymes. Thus, it is possible that the AAV2 protease function and those observed for the bee venom and bovine pancreatic PLA_2_ utilizes an alternate residue with a polar group as a nucleophile. The confirmation of a mechanistic model for this enzyme in AAV2 is thus awaiting a 3D structure not yet available.

## 4. Conclusions

This study shows that the VP1u region of AAV2 and AAV5 are responsible for protease function (Figure 1) in addition to the previously reported PLA_2_ function. For AAV2, metal chelation with EDTA or EGTA reduced the rate of protease activity but did not eliminate function, providing evidence that metal binding to the VP1u region of AAV2 is not the sole determinant for catalysis but likely plays an ancillary role by maintaining structural integrity of the domain (Figure 2). Reduction of activity by human A2M indicates that this protease function can be partially inhibited by a non-specific protein inhibitor but an effect on the viral life cycle remains to be elucidated, because pre-incubation of AAV2 with A2M increases cellular transduction in HEK293 cells (Figure 3). Observations with the susceptible substrates, for example, native casein, gelatin and heat denatured BSA, suggest that the target for the VP1u protease function are disordered proteins (Figure 4). These exist at different points in the parvovirus life cycle, including during nuclear entry. Interestingly, these studies also show that other PLA_2_ enzymes have proteolytic activity against casein, supporting a suggestion that the protease active site in AAV2 VP1u overlaps, in part, with the PLA_2_ domain (Figure 5). This suggestion is further supported by the reduction of protease function in the AAV2 PLA_2_ catalytic site variant ^76^HD/AN. However, the protease and PLA_2_ sites are not identical because H38, a non-PLA_2_ residue affects protease function (Figure 6). Moreover, while the AAV PLA_2_ activity is necessary for virion escape during the acidic conditions of the late endosome, the protease function described here operates at neutral pH, suggesting that the PLA_2_ and protease functions of the VP1u have different roles in the cellular context. The findings of this study highlight a convergent evolution of the PLA_2_ enzyme domain in active site sequence and structure and the acquisition of multiple enzyme functions within a single protein domain.
